# The Influence of Boron on the Structure and Properties of Hybrid Compounds Containing Zirconium and Phosphorus

**DOI:** 10.3390/gels8100667

**Published:** 2022-10-17

**Authors:** Petru Merghes, Gheorghe Ilia, Iosif Hulka, Vlad Chiriac, Narcis Varan, Vasile Simulescu

**Affiliations:** 1 Banat’s University of Agricultural Sciences and Veterinary Medicine ‘‘King Michael I of Romania’’ from Timisoara, Calea Aradului 119, 300645 Timisoara, Romania; 2 Faculty of Chemistry, Biology, Geography, West University of Timisoara, Pestalozzi Street 16, 300115 Timisoara, Romania; 3 Research Institute for Renewable Energies, Politehnica University Timisoara, Gavriil Musicescu Street 38, 300501 Timisoara, Romania

**Keywords:** sol–gel, organic–inorganic hybrid materials, boron, zirconium, phosphorus, inorganic polymers, SEM–EDX, TGA

## Abstract

In the present work, novel organic–inorganic hybrid materials containing boron, zirconium, and phosphorus were synthesized at different molar ratios, using the sol–gel method, starting from zirconyl chloride hexa-hydrate, triethyl borate, and phenyl phosphonic acid as the precursors. The sol–gel process is used for the first time in the present work in order to obtain organic–inorganic hybrids (or the so-called inorganic polymers) containing together boron, zirconium, and phosphorus. The sol–gel syntheses were performed at room temperature in ethanol. Zirconium containing compounds are already well known for their applications in medicine in restorative or prosthetic devices, including dental implants, knee and hip replacements, middle-ear ossicular chain reconstruction, and so on. Zirconium is a strong transition metal, which started to replace hafnium and titanium in the last decade in important applications. On the other hand, boron has the capability (similar to carbon) to form stable covalently bonded molecular networks. In addition to this capability, boron also offers mixed metallic and nonmetallic properties, because of its place on the periodic table, at the border between metals and nonmetals. Boron is responsible for the higher thermal stability of synthesized hybrid compounds. In the structure of those hybrid compounds, zirconium, boron, and phosphorus atoms are always connected via an oxygen atom, by P-O-Zr, Zr-O-Zr, or Zr-O-B bridges.

## 1. Introduction

The sol–gel process is a green technique of great interest nowadays for the synthesis of new hybrid materials containing phosphorus. Moreover, the sol–gel method involves green solvents and mild conditions, such as low temperatures (usually the sol–gel syntheses occur at room temperature). The sol–gel method is, among others, a promising route for the synthesis of metal phosphonates as metal organic frameworks (MOFs). It has two steps: first, the sol formation and, furthermore, the transition to the gel phase. The sol phase is a colloidal suspension of solid particles in a liquid, and the gel phase is a gelatinous network represented by a dispersion of a liquid in a solid. In other words, the sol–gel process represents the conversion of monomers into a colloidal solution (sol), which is the precursor for obtaining a network (gel) afterwards. Sol represents a solid dispersed in a liquid, and gel is a liquid dispersed in a solid. By using the sol–gel method, it is possible to introduce a large variety of organic moieties into an inorganic matrix. Organic–inorganic hybrid materials with different functional groups (alcoholic, carboxylic, sulphonic, etc.) have already been prepared using this method, and their proton conduction properties have been investigated [1,2,3,4].

The presence of the structural hydroxyl protons [2,3,4] indicate a good potential for these materials to exhibit solid state proton conduction [1,5,6,7]. Discovering new proton conductors is an area of great interest nowadays because of the potential use of those compounds for different applications, such as in sensors, water electrolysis units, and in other common electrochemical devices. By using the sol–gel process or by grafting on different surfaces, several hybrid compounds could be obtained, such as organic–inorganic networks, including phosphorus containing materials such as phosphonate metal organic frameworks, heterocyclic compounds, materials with flame retardant properties, and grafted materials for further use as new compounds with promising applications in electrochemical devices, as well as in the fields of catalysis, medicine, or agriculture [1,8,9,10,11,12,13,14,15,16,17,18,19].

Therefore, because of their structures, the hybrid materials can be obtained from organic–inorganic transition metal networks (supramolecular structures) [20,21,22,23,24,25,26,27]. Such materials contain P-O-Zr, Zr-O-Zr, and Zr-O-B bridges on their molecular structure, as well as carbon from the organic moiety. All of these bonds between the heteroatoms are contained on the organic–inorganic hybrid compound, and form a complex structure that could be regarded as a macromolecule (so-called inorganic polymer), which is characterized by a high mass and by the so-called gyration radius, as in the case of biopolymers, for instance in proteins [28,29]. From the dependence of radius gyration vs. molar mass, for a polymer, the conformation could be obtained. Unfortunately, this is not the case also for those organic–inorganic hybrids, because their structure could not be easily predicted (at least, not as simply, as in the case of a polymer or co-polymer, with one or two monomer units which are constantly repeated). Moreover, the determination of the conformation, in the case of biopolymers, is possible by using SEC-MALLS techniques. This method is applicable only for soluble compounds, but MALLS alone could also be used for insoluble materials.

Hybrid materials with a rigid inorganic backbone and flexible organic part, with groups and functionalities that bear protons (such as -OH, -COOH, and -SO_3_H), have already been synthesized [7]. Therefore, the sol–gel method is a promising route for obtaining several classes of compounds of great interest for many applications, as described above. Its main advantage is that it represents a green alternative that could replace some of the classical syntheses, in order to decrease the negative effects on the environment.

The novelty of the present work is the synthesis and characterization of organic–inorganic hybrid compounds containing boron, zirconium, and phosphorus, starting from zirconyl chloride hexa-hydrate, phenyl phosphonic acid (PPA), and triethyl borate, using the sol–gel method as well as the IR, TGA, SEM, and EDX techniques. Moreover, this is the first-time boron has been used together with zirconium and phosphorus for obtaining such organic–inorganic hybrid polymer materials using the sol–gel synthesis.

## 2. Results and Discussion

The organic–inorganic hybrid materials (**S1**–**S4**) obtained in the present work were synthesized using the sol–gel process in ethanol for six hours at room temperature. The starting reagents of PPA, ZrOCl_2_·6H_2_O, and B(OEt)_3_ were used at different molar ratios, as described in Table 1.

When added, B(OEt)_3_ slowly hydrolyzed and boric acid was obtained during the synthesis. Therefore, by using triethyl borate, boric acid was continuously obtained and in situ generated during the sol–gel process. In other words, the sol–gel chemical reaction took place with boric acid, but B(OEt)_3_ was used in order to decrease the reaction speed. If boric acid was used directly from the beginning of the sol–gel process (and not triethyl borate), the reaction would be too fast and the obtained yield would significantly decrease.

The obtained metal–organic frameworks were not soluble in ethanol or in water. This change in solubility, in comparison with the reagents and precursors used, was actually the first indication that the reactions took place—the reagents were soluble and the product was not. During the reaction, the product separated as a sediment at the bottom, while the reagents (even if in excess or not reacted yet) were still in the solution and dissolved in ethanol. The thickness of the sediment layer containing the insoluble inorganic polymer increased over time until a certain moment, when the synthesis was finished. Therefore, because of this change in solubility during the sol–gel process, once obtained, the product was found on the bottom of the beaker as a sediment because of its insolubility and higher density. The interface between the sediment containing the hybrid and the solvent, which could still contain unreacted reagents (if in excess), would make the next steps of separation and purification of the product easier.

Therefore, after six hours, the obtained products were further washed and filtered several times, and then dried for other six hours in an oven at a temperature of 80 °C. In comparison with our previous research on the synthesis of hybrid compounds (starting from phosphonic acids), as well as in comparison with state-of-the-art metal–organic frameworks (MOFs) and hybrid materials fields [20,21,24,25,30], the main novelty of the present work is that the use of boron, which is embedded in the structure of the hybrids, provided a higher thermal stability. The boron changed and improved the properties of the novel synthesized materials. The synthesized hybrids led to obtaining organic–inorganic transition metal networks, which can be regarded as MOFs [17,18,19,20,21], in the case of the present work containing P-O-Zr, Zr-O-Zr, and Zr-O-B bridges. Nevertheless, it should be pointed out that those bridges could only be obtained in the mild conditions used for the sol–gel process. On the other hand, the phenyl groups did not react and therefore were found also on the structures of the hybrids, connected at the phosphorus atom, as in the structure of phenyl phosphonic acid (PPA). The organic–inorganic hybrid compounds had a 3D structure, with phenyl (*Ph*) connected to the phosphorus atom, as in PPA, and then with randomly long chains of Zr, P, and B (not necessarily in this order, or at least not always) connected by the above-mentioned bridges containing oxygen in between. This complex structure can be regarded as an inorganic polymer, or so-called MOF, because it is actually a network that can be developed in several directions and for this reason, we can say it is a 3D system.

**Figure 1:**
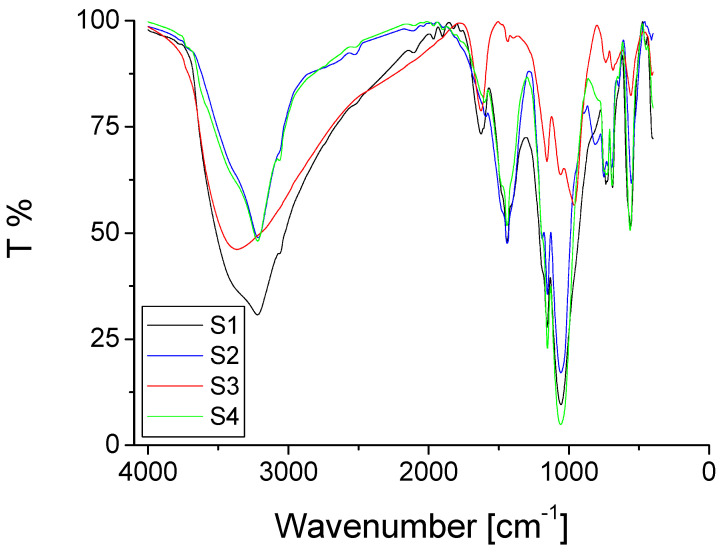
IR results obtained for compounds **S1**–**S4**.

Such hybrid polymer materials are suitable to form supramolecular structures. The IR spectra (Figure 1) showed that the hybrid **S2**, obtained when B(OEt)_3_ was in excess (Table 1) and the hybrid **S4** that was synthesized when phenyl phosphonic acid was in excess, had more similar structures than the others.

Moreover, their structures (**S2** and **S4**) were different than the structures obtained for the hybrid compounds **S1** and **S3** (obtained at equal molar ratio or when ZrOCl_2_ was in excess), according to the IR data (Figure 1). Hybrids **S2** and **S4** showed relatively similar IR results, because of a 2:1 molar ratio of boron or PPA related to zirconium, which did not significantly change their structures. However, this does not mean that all of their structures are exactly the same. All of the synthesized materials were different from each other, but their properties were similar, as expected, because they were synthesized in the same way, by using same method and slightly different molar ratios for the same reagents and precursors.

From all four of the obtained hybrid compounds containing phosphorus, zirconium, and boron, the one that showed significant differences in the second part of the IR graph, between 2000 and 500 cm^−1^, was compound **S3** synthesized in the presence of excess ZrOCl_2_. Because of the excess of ZrOCl_2_, this material was expected to have more Zr-O-Zr bridges in comparison with the **S1**, **S2**, and **S4** hybrids. Actually, hybrids **S1**, **S2**, and **S4** were expected to have a much lower number of Zr-O-Zr bridges, because zirconium was used in a lower amount in those compounds. Therefore, zirconium should be involved first in P-O-Zr and B-O-Zr bridges, because of excess phosphorus and/or boron. The repeating units of PPA, zirconyl chloride, and boron, and the formation of the bridges between zirconium, boron, and phosphorus, as previously mentioned, on the synthesized organic–inorganic hybrid compounds, prove that their structures could actually be regarded as co-polymers. Such compounds are also commonly named inorganic polymers. All of those materials **S1–S4** synthesized in the present work showed no solubility, as expected for many synthesized polymers in general.

**Figure 2:**
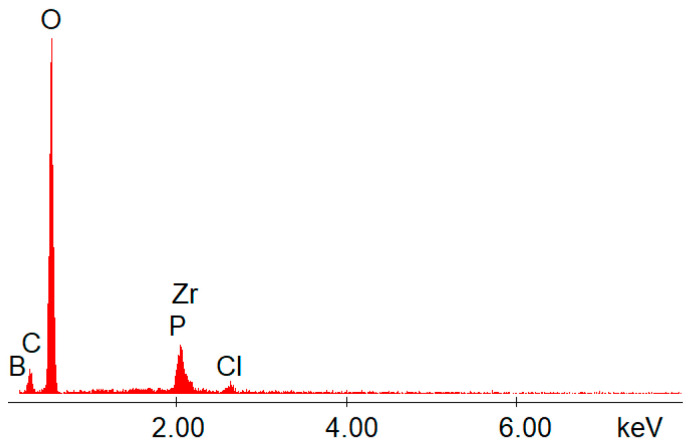
EDX data for the hybrid compound **S1**.

**Figure 3:**
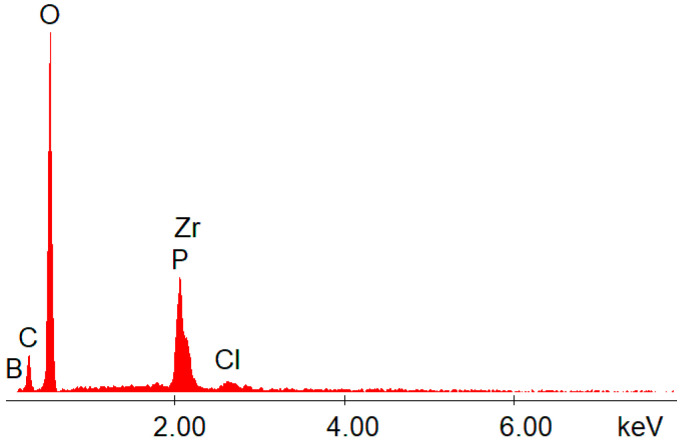
EDX data for the hybrid compound **S3**.

The EDX data (Figure 2 and Figure 3) confirmed the presence of boron, as well as the presence of zirconium and phosphorus, on the structures of the synthesized hybrids.

The IR and EDX analyses proved that the chemical reactions performed using the sol–gel method took place, in addition to the observed changes in solubility, and thus different materials were obtained. The SEM–EDX methods involved in this study made no significant difference on the spectra between P and Zr, or C and B. Therefore, those peaks were too close to distinguish between them in Figure 2 and Figure 3, but for each EDX graph, a table was also attached after the measurement (Table 2).

In Table 2, it is clear which element is represented on the EDX spectrum. It is also easy to distinguish the signal intensity (according to the amount/i.e., ratio) for each element detected by EDX. Other important information observed from the EDX measurements (Table 2 and Figure 2 and Figure 3) was the high amount of oxygen in the structure of the hybrids. This actually confirmed that the synthesized inorganic polymers contained a lot of P-O-Zr, Zr-O-Zr, and Zr-O-B bridges (and obviously oxygen was involved in all of those bridges). By using the sol–gel method, mild conditions were involved. These mild conditions could not lead to obtaining direct bonds (without oxygen), such as P-P, P-Zr, Zr-Zr, Zr-B, or B-B. These examples were not possible, and as a consequence, oxygen was involved in all of the bridges formed between the heteroatoms from the hybrid’s structure. All of the metal–organic frameworks (MOFs) synthesized here contained a high amount of oxygen (more than 50%).

**Figure 4:**
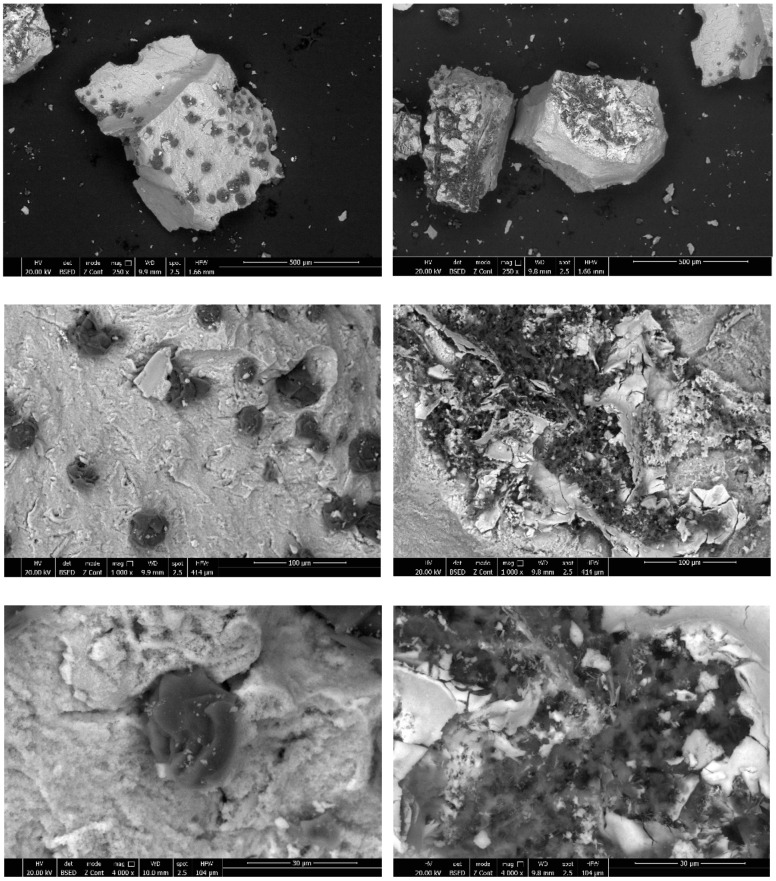
SEM images of the hybrid compound **S1**.

In addition to the IR and EDX methods, the synthesized hybrids were also analyzed by using SEM and TGA techniques. The SEM images (Figure 4) showed a similar morphology containing compact structures, generally bigger than 100 μm.

**Figure 5:**
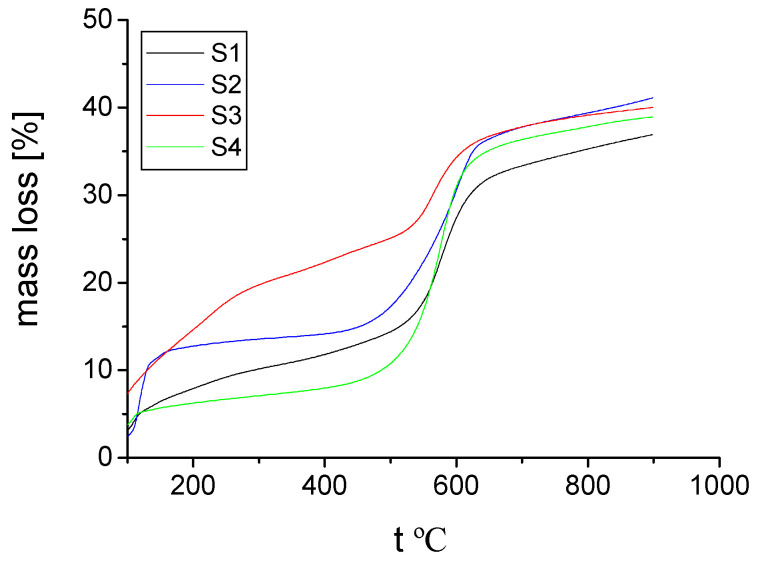
Mass loss (%) resulted from TGA in air for the synthesized hybrid compounds **S1–S4**.

**Figure 6:**
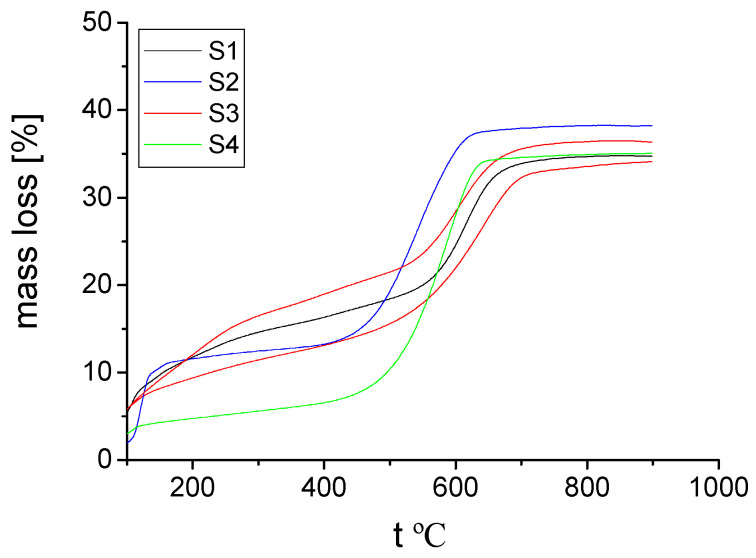
Mass loss (%) that resulted from TGA in nitrogen for hybrid compounds **S1**–**S4**.

In addition, the thermal analysis (TGA) proved the high thermal stability of the hybrid materials **S1**–**S4** (Figure 5 and Figure 6). All of the hybrids synthesized in the present work behaved relatively similarly in air and in nitrogen, from a thermal stability point of view.

The amount used for TGA measurements in order to determine the thermal stability ranged between 4.5 and 6 mg. Until reaching a temperature of 500ºC, in air, hybrid **S3** lost around 25% of its initial mass (Figure 5). The other hybrids were more stable in this temperature range (the hybrid **S4**, for example, showed a mass loss below 10%). From 500 °C to 600 °C, the mass loss increased up to 30–35% for all of the synthesized hybrids, in air and in nitrogen (Figure 5 and Figure 6).

In both air and nitrogen, the mass loss of the **S1**–**S4** hybrids reached its maximum, slightly below 40%, up to a temperature of 900 °C. Actually, in nitrogen, this maximum mass loss was reached just below 700 °C, and further, from 700 °C to 900 °C, almost no mass loss was observed. This all proves that the synthesized hybrid materials are suitable for different applications at high temperatures, especially until 500 °C.

The hybrid compound **S4**, obtained when PPA was in excess, was the most stable until 500 °C, from all of the compounds synthesized in the present work. At 900 °C, the thermal stability was more or less similar for all **S1**–**S4** hybrids, as already mentioned. If we compare the TGA data of hybrids **S1**–**S4** with the results of the organic–inorganic hybrid materials synthesized in our previous work [30], also starting from phosphonic acids, we can say that the thermal stability increased in the presence of boron. This higher thermal stability was mainly as a result of boron’s capability to form stable covalently bonded molecular networks, as well as its mixed metallic and non-metallic properties.

Such hybrids compounds containing zirconium, boron, and phosphorus could have potential applications in different fields, such as medicine, supramolecular chemistry, or for different electrochemical devices, by combining the effects of those elements. The human body uses boron for building strong muscles and bones, for preventing osteoarthritis, and for improving thinking skills and muscle coordination. Therefore, boron brings significant advantages in the structures of the obtained hybrid compounds, including a higher thermal stability. On the other hand, zirconium containing compounds are well-known for their applications in medicine as restorative or prosthetic devices, including dental implants, knee and hip replacements, middle-ear ossicular chain reconstruction, and so on. Moreover, zirconium binds urea and this property was already used for the patients with kidney disease [15]. Zirconium is also important for different imaging techniques used in medicine. The isotope ^89^Zr has been used, for example, for the tracking and quantification of molecular antibodies with positron emission tomography cameras (PET) [31,32,33,34].

## 3. Conclusions

In the present work, novel hybrid materials containing zirconium, boron, and phosphorus compounds were synthesized using the sol–gel method, starting from zirconyl chloride hexa-hydrate (ZrOCl_2_·6H_2_O), triethyl borate B(OEt)_3_, and phenyl phosphonic acid (PPA) as the reagents. All of the syntheses were performed at room temperature in ethanol, at different molar ratios. These organic–inorganic hybrids have complex structures and form transition metal networks containing Zr-O-Zr, Zr-O-B, and Zr-O-P bridges. The phenyl groups did not react and therefore were also found on the structures of the synthesized hybrids, connected at the phosphorus atom, as in the structure of PPA. Phosphorous, zirconium, and boron atoms are always connected via an oxygen atom on the structure of the hybrids. In the mild conditions used in the sol–gel process, it is not possible to obtain P-O-P, P-O-B, or B-O-B bridges. The organic–inorganic hybrid compounds showed a 3D structure, with phenyl (*Ph*) connected directly to a phosphorus atom, as in PPA, because the phenyl groups did not react in this case. The hybrid materials also contained randomly long chains of Zr, P, and B connected by the mentioned bridges that always contained oxygen in between. This complex structure can be regarded as an inorganic polymer, or so-called MOF, because actually it is a network developed in several directions (obviously it has no linear molecular geometry).

The obtained hybrids were analyzed using IR, TG, EDX, and SEM methods. The hybrid **S1** showed more differences in the first part of the IR graph (between 4000 and 2000 cm^−1^) in comparison with the other obtained compounds. It was synthesized at equal molar ratios for all of the reagents used. From all four of the obtained hybrids containing phosphorus, zirconium, and boron, the one that showed more differences in the second part of the IR graph (between 2000 and 500 cm^−1^) was compound **S3**, synthesized in the presence of excess ZrOCl_2_. The second part was more influenced by the PPA and zirconyl chloride. As a consequence of excess ZrOCl_2_, the hybrid material **S3** was expected to have more Zr-O-Zr bonds in comparison with the **S1**, **S2**, and **S4** hybrids. Actually, the hybrids **S1**, **S2**, and **S4** were expected to have almost no Zr-O-Zr bonds, because zirconium is not in excess in those compounds and thus it should be involved mostly in P-O-Zr and B-O-Zr bridges. The EDX data confirmed the presence of boron, as well as the presence of zirconium and phosphorus on the structures of the organic–inorganic hybrids. The SEM images showed a morphology containing compact structures, with sizes, in general, bigger than 100 mm. In addition, for all of the hybrid polymer materials (**S1–S4**) synthesized in the present work, the EDX data confirmed the presence of boron, zirconium, and phosphorus.

The obtained materials were not soluble in ethanol or in water. In addition, they showed a high thermal stability in both air and nitrogen. From the TGA results, until reaching a temperature of 500 °C, the most stable was hybrid **S4**, with a mass loss below 10%. At 900 °C, all of the synthesized **S1**–**S4** hybrids showed more or less similar mass loss, slightly below 40%. This is first time that boron, phosphorus, and zirconium (as well as carbon from PPA as a source) have been used together in a hybrid sol–gel synthesis for obtaining organic–inorganic hybrids. To have carbon and boron together into the hybrid polymer molecule, creates more alternatives for applications, because both carbon and boron have the capability to form stable covalently bonded molecular networks. Because of their stability, such organic–inorganic networks are expected to form supramolecular structures and to have potential applications in catalysis, medicine, energy storage, electrochemical devices, sensors, and supramolecular chemistry.

These compounds showed a similar behavior and nearly the same morphology observed from the SEM images, a similar composition observed from EDX, and also quite a similar high thermal stability, but all of those synthesized materials were still different from each other because of the different molar ratios of the starting reagents and precursors used.

## 4. Methods and Materials

Zirconyl chloride hexa-hydrate (ZrOCl_2_·6H_2_O), phenyl phosphonic acid (PPA), and triethyl borate B(OEt)_3_ were purchased from Fluka. All of the reagents used in the present work where ethanol was also used as a solvent, were of high purity. Zirconyl chloride (also known as zirconium oxychloride or zirconium dichloride oxide) is commonly synthesized by the hydrolysis of zirconium tetrachloride or by the reaction between zirconium oxide and HCl [22,23]. Phenyl phosphonic acid has already been used already as self-assembled monolayers in order to make an interface between organic semiconductors and transparent conductive oxides, or to functionalize titania particles [24,25].

Triethyl borate (found to also be named as boric acid triethyl ester, boron ethoxide, or triethoxyborane in the literature) is a weak Lewis acid [27], which burns with a green flame. Because of these properties, solutions of triethyl borate in ethanol are used in special effects and pyrotechnics. Triethyl borate can be obtained by the esterification reaction of boric acid and ethanol, in the presence of an acid catalyst [26,27]. Through the decomposition reaction, from triethyl borate, boric acid is obtained.

The sol–gel method was used for obtaining the hybrid polymer materials containing zirconium, boron, and phosphorus. For the determination of the material structures, several analysis methods were employed, such as IR, TG, SEM, and EDX. A Jasco FT-IR 4200 Spectrometer was used for recording the IR spectra. Thermal analysis (TGA) was carried out by changing the temperature between 20 °C and 900 °C, by using an 851-LF 1100-Mettler Toledo apparatus in airflow. Its sensitivity was as low as 1 μg. The maximum amount of the sample, which could be measured, was 5 g. SEM and EDX characterizations were performed in vacuum, for solid samples of the obtained hybrids, by using a Jeol JSM 6400 Scanning Microscope coupled with an X-ray microanalyzer EXL II System Link Analytical, with a detector of 133 eV. The solid dry samples were analyzed as they were obtained after washing and drying, without sputtering them. The sputtering (a thin film deposition process) was not necessary, because the materials exhibited a good conductivity, which also led to obtaining SEM images of good resolutions and good quality.

## Figures and Tables

**Table 1:** Molar ratios of the reagents used for obtaining hybrid compounds **S1**–**S4**.

Synthesis	PPA	ZrOCl_2_	B(OEt)_3_
**S1**	1	1	1
**S2**	1	1	2
**S3**	1	2	1
**S4**	2	1	1

**Table 2:** EDX results for the hybrid compounds **S1** and **S3**.

Element (Wt%)	S1	S3
B	2.67	4.53
C	13.99	17.05
O	77.28	67.17
P	2.21	1.76
Zr	3.47	9.48

## Data Availability

The data presented in this study are available from the corresponding author upon request.

## Aims and Object

The main aim of the present work is to synthesize novel organic–inorganic hybrid materials in the form of metal–organic frameworks (MOFs), with a 3D structure containing boron, zirconium, and phosphorus. One of the goals is to observe the influence of boron on the structure, properties, and behavior of those hybrid compounds (also called inorganic polymers). The sol–gel method is used for obtaining hybrids at different molar ratios, in mild conditions, by using a green solvent with a low toxicity (i.e., ethanol). IR, SEM–EDX, and TGA methods were used for the analysis and characterization of the synthesized materials.
